# ToNER: A tool for identifying nucleotide enrichment signals in feature-enriched RNA-seq data

**DOI:** 10.1371/journal.pone.0178483

**Published:** 2017-05-25

**Authors:** Yuttachon Promworn, Pavita Kaewprommal, Philip J. Shaw, Apichart Intarapanich, Sissades Tongsima, Jittima Piriyapongsa

**Affiliations:** 1 National Center for Genetic Engineering and Biotechnology (BIOTEC), National Science and Technology Development Agency (NSTDA), Pathum Thani, Thailand; 2 National Electronics and Computer Technology Center (NECTEC), National Science and Technology Development Agency (NSTDA), Pathum Thani, Thailand; National Institutes of Health, UNITED STATES

## Abstract

**Background:**

Biochemical methods are available for enriching 5′ ends of RNAs in prokaryotes, which are employed in the differential RNA-seq (dRNA-seq) and the more recent Cappable-seq protocols. Computational methods are needed to locate RNA 5′ ends from these data by statistical analysis of the enrichment. Although statistical-based analysis methods have been developed for dRNA-seq, they may not be suitable for Cappable-seq data. The more efficient enrichment method employed in Cappable-seq compared with dRNA-seq could affect data distribution and thus algorithm performance.

**Results:**

We present Transformation of Nucleotide Enrichment Ratios (ToNER), a tool for statistical modeling of enrichment from RNA-seq data obtained from enriched and unenriched libraries. The tool calculates nucleotide enrichment scores and determines the global transformation for fitting to the normal distribution using the Box-Cox procedure. From the transformed distribution, sites of significant enrichment are identified. To increase power of detection, meta-analysis across experimental replicates is offered. We tested the tool on Cappable-seq and dRNA-seq data for identifying *Escherichia coli* transcript 5′ ends and compared the results with those from the TSSAR tool, which is designed for analyzing dRNA-seq data. When combining results across Cappable-seq replicates, ToNER detects more known transcript 5′ ends than TSSAR. In general, the transcript 5′ ends detected by ToNER but not TSSAR occur in regions which cannot be locally modeled by TSSAR.

**Conclusion:**

ToNER uses a simple yet robust statistical modeling approach, which can be used for detecting RNA 5′ends from Cappable-seq data, in particular when combining information from experimental replicates. The ToNER tool could potentially be applied for analyzing other RNA-seq datasets in which enrichment for other structural features of RNA is employed. The program is freely available for download at ToNER webpage (http://www4a.biotec.or.th/GI/tools/toner) and GitHub repository (https://github.com/PavitaKae/ToNER).

## Introduction

Understanding of RNA structure and function has been enhanced greatly in recent years through high-throughput RNA sequencing (RNA-seq). In RNA-seq, RNA is fragmented and converted to DNA libraries with adapter sequences incorporated for sequencing. Analysis of RNA-seq data is challenging because RNA transcripts vary widely in abundance. RNA-seq data are thus sparse for many transcripts of low to moderate abundance. Furthermore, RNA exhibits a variety of structural features such as 5′ capping, base editing and methylation that are important for function [1]. These features cannot be inferred directly from RNA-seq data as this information is lost during the conversion to DNA for library construction. The limitations of RNA-seq mean that RNA fragments containing the feature of interest must be enriched for transcriptomic identification. Features of interest can be identified as nucleotides with an over-abundance of aligned reads from the feature-enriched RNA-seq data. It is difficult to determine what level of enrichment is significant given the high complexity of RNA-seq data. For this reason, an unenriched control RNA-seq dataset is required for statistical analysis of the enrichment.

One of the most widely studied RNA structural features is the 5′ end nucleotide, which defines a gene’s transcription start site (TSS) and the core promoter element in close proximity that controls transcription. It is challenging to identify transcript 5′ ends directly from RNA-seq data by reconstructing transcript structures from overlapping reads aligned to the genome. This is because read coverage is not uniform along each gene owing to RNA fragmentation bias, in which the 5′ and 3′ ends of the RNAs are typically under-represented [2]. For many transcripts of low to moderate abundance, the sparse data of transcript ends are often not contiguous with the transcript body. Furthermore, transcripts with alternative 5′ ends may exist owing to transcription from alternative promoters or post-transcriptional processing. For these reasons, unambiguous mapping of 5′ ends is only possible using RNA-seq methods that enrich for the 5′ end RNA fragments.

In prokaryotes, transcript 5′ ends can be mapped using data from differential RNA-seq (dRNA-seq) [3]. In dRNA-seq, total RNA is treated with a 5′-phosphate-dependent exonuclease to deplete rRNA and other RNA species lacking a 5′ triphosphate cap. This depletion strategy results in enrichment for mRNA containing a 5′ triphosphate cap, which is resistant to the exonuclease. The enrichment employed in dRNA-seq is inefficient, since irrelevant RNA species with extensive secondary structure, e.g. tRNA are also resistant to exonuclease. Recently, the Cappable-seq method was described as an alternative transcript 5′ end mapping technique, which employs a more efficient enrichment method [4]. In Cappable-seq, a biotinylated guanosine cap is added specifically to the 5′ triphosphate end of mRNA using vaccinia virus capping enzyme. The biotinylated moiety is used as a purification handle to highly enrich for transcript 5′ ends, taking advantage of the high affinity interaction of biotin with streptavidin.

In these transcript 5′ end mapping methods, unenriched RNA is sequenced in addition to 5′ end-enriched RNA. The unenriched RNA serves as the control, and hence enrichment for each nucleotide position can be quantified using the paired library data. Algorithms have been developed for quantifying enrichment from dRNA-seq data for the purpose of annotating transcript 5′ ends. The TSSpredator algorithm described in [5] is a tool for filtering candidate nucleotides based on arbitrary pre-selected thresholds of normalized enrichment ratios across experimental replicates. The normalization process corrects for systematic variations in enrichment efficiency from one experiment to another by globally rescaling enrichment ratios. However, since no statistical modeling of the enrichment is performed, there is no way to assess confidence in the annotations, which may be too conservative or too lenient depending on experimental variation of the enrichment. The TSSer algorithm evaluates posterior enrichment probabilities in local genomic regions using information of standardized enrichment ratios across replicate experiments [6]. The posterior probabilities may be unrealistic though if there is substantial variation in enrichment efficiency across experiments.

The TSSAR algorithm [7] is currently the only available tool for determining the statistics of enrichment at each nucleotide position under a frequentist model. TSSAR fits the data to the Skellam model distribution (the difference in zero-inflated Poisson distributions of read counts between unenriched and enriched datasets). The model must be applied in local genomic contexts to account for variation in transcript abundance, and in some regions the data fit poorly or cannot be modeled. Therefore, some sites may be falsely identified and some true sites may be missed because of inappropriate model parameters. Although data of unenriched background are available for Cappable-seq, no algorithm has been specifically developed for analysis of Cappable-seq data that utilizes the control data for statistical modeling. In [4], candidate enriched positions were identified using a threshold of normalized read counts from the enriched Cappable-seq dataset, and no statistical modeling of enrichment was used to assess the confidence of annotations. In theory, the tools developed for dRNA-seq analysis could be applied for Cappable-seq data; however, the more pronounced bias in read count distribution in Cappable-seq enriched data (owing to the greater enrichment efficiency) compared with dRNA-seq could affect algorithm performance.

Although the transcript 5′ end has received the most attention, enrichment methods have been developed to study other RNA structural features such as *N*^6^-methyladenosine (m^6^A) [1]. However, very few enrichment methods have been applied for mapping these modifications at the transcriptomic scale. As we learn more of RNA structure and function, it is likely that new biochemical methods for specifically enriching different RNA features with greater efficiency will be developed. The analysis of RNA-seq datasets enriched for different features would be simplified if a common analytical procedure could be employed. Moreover, the analysis should use a simple statistical framework that models data distributions appropriately, irrespective of the enrichment method or transcriptomic complexity. We propose an alternative approach for obtaining statistics of nucleotide enrichment by fitting the data to a global distribution. Using this framework, enrichment statistics can be combined across experimental replicates for improved reproducibility and power to detect features of interest.

In this paper, we developed a tool which we call ToNER (Transformation of Nucleotide Enrichment Ratios) for annotating RNA features by analysis of RNA-seq data obtained from enriched and unenriched control libraries. The tool reports statistics of enrichment for all nucleotides using a global distribution model. The tests with Cappable-seq show the high power of ToNER to detect the annotated TSSs, in particular when combining data from experimental replicates. The potential to apply ToNER to detect other biologically relevant RNA features is also demonstrated.

## Materials and methods

### Overview of the ToNER tool

**Fig 1:**
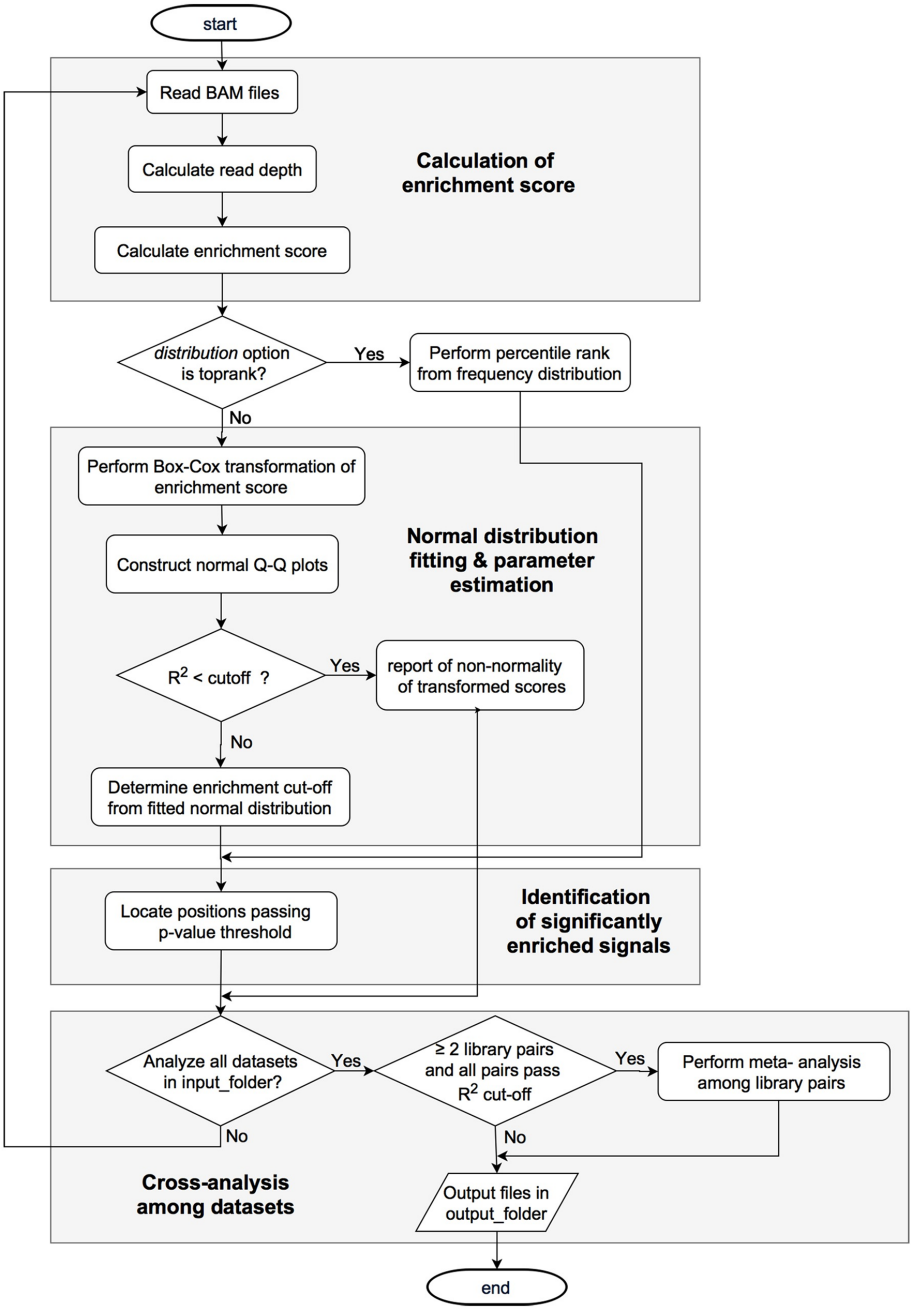
The workflow of the ToNER software.

Fig 1 outlines the detailed workflow of the tool. In summary, RNA-seq data aligned to a reference genome are provided in Binary Alignment Map (BAM) format as inputs. These BAM files comprise enriched and unenriched (control) library datasets. The analysis starts by calculation of position-wise normalized read depth ratio between two libraries, referred to as the enrichment score, for all mapped genome positions. The genome-wide enrichment scores are then transformed and fitted to the normal distribution by the Box-Cox procedure [8]. The modeled distribution parameters are reported and, hence, p-values of the enrichment can be determined for each nucleotide. Finally, significantly enriched positions that pass a p-value cutoff are identified. If data from multiple enrichment experiments are provided, meta-analysis across replicate datasets is employed to increase power to detect significantly enriched positions. More details of each work step are described in the following sections and are also summarized on the ToNER webpage (http://www4a.biotec.or.th/GI/tools/toner-project/TSSrr.png).

### Enrichment score calculation

RNA-seq data from an RNA-enriched library and a separate control library are preprocessed, i.e., removal of adapter, poor quality reads and trimming poor-quality bases from the ends. The preprocessed data are aligned to a reference genome sequence using standard tools such as Bowtie2 [9] which provide the aligned data in BAM format. The choice of data preprocessing and alignment tool is dependent on the data analysed. For example, eukaryotic data require alignment using splice-aware tools such as TopHat2 [10]. In general, we think that stringent mapping that considers only uniquely mapped reads is preferable, since ambiguously mapped reads could skew the distribution fitting of enrichment scores. ToNER does not require paired-end read data, although if paired-end data are available, users could choose to preprocess the mapped reads so that only the properly paired reads are employed. This would increase the stringency of read mapping and eliminate potentially mis-aligned forward reads used for distribution fitting. ToNER accepts BAM files from enriched and unenriched control libraries. Positions without mapped reads in both enriched and unenriched libraries are excluded from the analysis. The read depth value, which refers to the total number of reads (bases) aligned at a given genome position, is calculated separately for two RNA-seq libraries for all mapped positions in the genome. In a typical RNA-seq dataset, most positions will have low depth values, including sites of biological interest. Therefore, by default, all genomic positions with at least one mapped read from either of the RNA-seq libraries are included in the analysis. To avoid division by zero for positions that have mapped reads in only one library, a pseudo-read equal to one count is added to all positions in both libraries. Although the analysis is typically inclusive of all transcribed positions, it may be desirable to exclude positions with low depth values in one or both libraries. The user can specify minimum read depth in either library, or sum of both libraries for a position to be considered for analysis. Moreover, users can also select the non-pseudo count option if minimum read depth filters of one or greater in both libraries are employed. The software also allows users to limit analysis of enriched nucleotides to only the read start or end position, as is required for study of transcript ends. Alternatively, all read positions can be selected for analysis.

After obtaining read depth values for all mapped nucleotides, the enrichment score for each nucleotide is calculated. The enrichment score is defined as the ratio of the read depth value of enriched library to the read depth value of unenriched library normalized to total read depth of all positions. The enrichment score for a nucleotide i^th^ (E_i_) is thus calculated as:

$$E_{i}=\frac{D_{1i}}{D_{2i}}\times \frac{\sum_{i=1}^{n}D_{2i}}{\sum_{i=1}^{n}D_{1i}}$$

where D_1i_, D_2i_ are the read depth values on the position i^th^ for the enriched library (1) and the unenriched library (2), respectively and *n* is the total number of genomic positions with read depth values.

### Fitting data to the normal distribution

We assume the null hypothesis that if there is no enrichment, the scores will be normally distributed. The enrichment scores in the experimental data comprise the majority of positions that are not enriched and a minority of enriched positions. The latter population skews the distribution. In order to obtain normally distributed data, a transformation is required to mitigate the skew. The enrichment score data are transformed using the Box-Cox transformation procedure [8]. The mean (μ) and standard deviation (σ) parameters can be determined from the transformed data. A quantile-quantile plot (Q-Q plot) is used to assess the goodness of fit of the transformed data to the normal distribution. Normal Q-Q plots are constructed for enrichment scores before and after data transformation for comparison. The goodness of fit is assessed by the program using an R^2^ cutoff, the default of which is 0.90. If the data pass the R^2^ normality test, the critical value threshold of significant enrichment score is determined from the fitted normal distribution according to a user-defined p-value cutoff (default p = 0.05). In case the transformed data do not pass the R^2^ threshold, users can adjust the R^2^ cutoff or use the percentile rank option. In this option, enrichment scores are ranked and positions with the top 5% of the enrichment scores (95^th^ percentile cutoff) are automatically selected and reported as a list of putatively enriched positions, although no p-values of enrichment are reported. The percentile rank cutoff is also user-adjustable.

### Identification of significantly enriched positions

Significantly enriched positions are reported by the program with details of the read depth value in each RNA-seq library, the enrichment score, and the associated p-value of enrichment. The program also locates the enriched positions relative to genes if the gene annotation file in the General Feature Format (GFF) is provided along with the input BAM files. The distance from the enriched positions relative to the start position of the nearest downstream gene or end position relative to the nearest upstream gene can also be reported. ToNER also outputs the corresponding GFF file of the enriched positions, which enables users to conveniently process the results further using other tools such as genome browsers. Summary data statistics of the number of positions with mapped reads in either or both RNA-seq libraries are also provided. Moreover, the Q-Q plots and the details of distribution model parameters are displayed as Portable Network Graphics (PNG) files.

### Increasing detection power by meta-analysis

If data from more than one experiment are available, meta-analysis of enrichment statistics can be performed to increase power of detection. ToNER employs Fisher’s combined probability test to calculate combined p-values of enrichment from all experiments. Instead of Fisher’s combined test, the ‘consensus’ option can be selected to report enriched positions if they are significant in individual experiments among at least a minimum number of replicates specified by the user. The genomic positions passing the significance criterion are reported, together with p-values from individual experiments. Fisher’s combined p-value is also reported if this option is selected.

### Application of ToNER on RNA-seq datasets

We tested the ToNER tool for annotating transcript 5′ ends using Cappable-seq data of *Escherichia coli (E*. *coli)* [4]. Two enriched library replicates and a shared unenriched control library were analysed. Sequence reads from all datasets were downloaded from the European Nucleotide Archive (ENA) accession number PRJEB9717. Adaptor sequences were trimmed from the reads using cutadapt version 1.3 [11] with default parameters and–a AGATCGGAAGAGCACACGTCTGAACTCCAGTCAC. The reads were aligned to the *E*.*coli* strain K-12 genome (GenBank: U00096.2) using Bowtie2 with default settings [9]. We did not remove duplicates, as reads with the same start may derive from different RNA molecules, especially at biologically relevant transcript 5′ ends. The BAM files were used as input for ToNER, selecting read start positions only for analysis.

The significantly enriched positions called by ToNER from Cappable-seq data were compared with those identified by the standalone version of the TSSAR software [7]. TSSAR analysis was performed using the default window size of 1000 nt, minimum read depth of one (—minPeak 1), no peak clustering (—nocluster), and p-value cutoff of one (reporting p-values of all positions). Fisher’s combined p-values were calculated from the p-values reported by TSSAR for individual experiments using the scipy.stats.combine_pvalues function in the Python SciPy package.

For recall analysis of known transcript 5′ ends from Cappable-seq data, annotated TSS in the RegulonDB database [12] were taken to be true 5′ ends. Only TSS positions annotated in the RegulonDB database subset I (manually annotated, with strong evidence from multiple experimental data types, including presence of canonical core promoter) were selected for recall tests. If significantly enriched positions are located within 5 nt from the closest annotated TSS, we considered this TSS position as being detected. Significantly enriched nucleotides may not correspond precisely to annotated TSSs for biological reasons, e.g. alternative transcription initiation, or for data analytical reasons such as trimming of the sequence reads in the data preprocessing step.

We also analyzed *E*.*coli* dRNA-seq datasets [13] using ToNER and TSSAR. Sequence reads from all datasets were downloaded from the Gene Expression Omnibus (GEO) database under accession number GSE55199. We used the same data preprocessing and alignment methods as in the original publication [13]. Briefly, reads were trimmed with a cutoff phred score of 20 by the programfastq_quality_trimmer from the FASTX toolkit (http://hannonlab.cshl.edu/fastx_toolkit/) version 0.0.13, and preprocessed reads were then mapped to the *E*. *coli* MG1655genome (NCBI: NC_000913.2) using READemption software [14].

The application of ToNER for annotating *N*^6^-methyladenosine (m^6^A) sites was tested on m^6^A-seq data reported in [15]. The data were downloaded from the NCBI Gene Expression Omnibus, series accession number GSE37003. Two enriched library replicates (IP1, IP2) and the unenriched control libraries (Input1, Input2) were analysed. Reads were preprocessed by sequence trimming of poor-quality base calls (N) and removing reads of low overall quality (average Phred quality score <20) using PRINSEQ [16]. Reads were then aligned to the hg18 reference genome using TopHat2 (unique mapping setting) [10]. The output BAM files were given as inputs for ToNER analyses using the default pseudo-count setting and analysis of all read positions.

## Results

### Evaluation of ToNER for transcript 5′ end mapping from Cappable-seq data

We tested the ToNER tool for annotating transcript 5′ ends using Cappable-seq data [4]. The program output of frequency distributions of the number of mapped read starts per position of the two compared libraries reveal a lower complexity of enriched libraries, which are largely consistent with the effective enrichment of RNA 5′ end fragments employed in this method (S1 Fig). Furthermore, only 15 and 17% of positions have mapped reads in both enriched and unenriched control libraries for experimental replicate 1 and replicate 2, respectively (S1 Fig). Therefore, read depth values for most positions are sparse, and if a filter of minimum read depth is employed to exclude positions that lack depth values in the unenriched library, genuine transcript 5′ ends may be missed.

**Fig 2:**
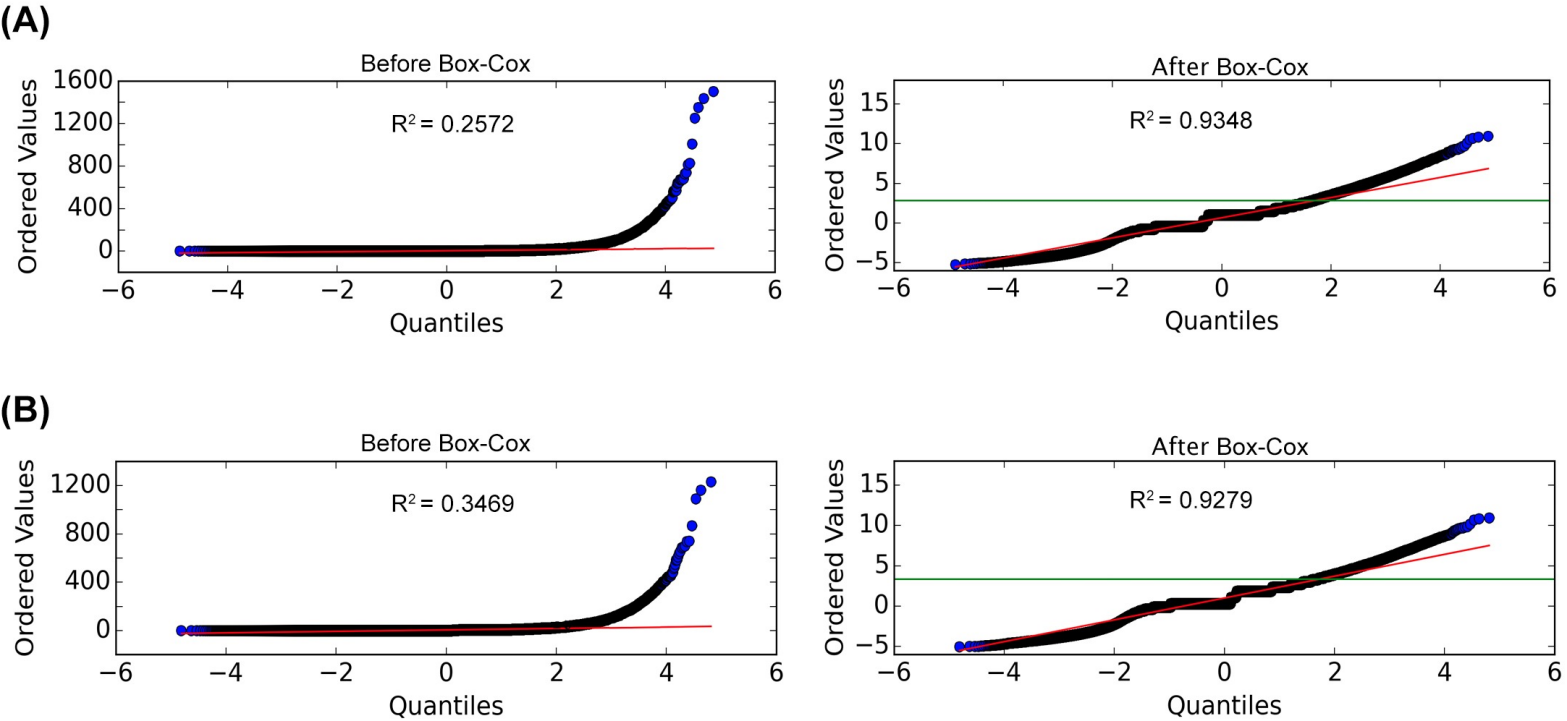
The normal Q-Q plots of the calculated enrichment scores from Cappable-seq data. Q-Q plots of enrichment score quantiles calculated from Cappable-seq data (vertical axes) versus normally distributed theoretical quantiles (horizontal axes) are shown for scores before and after Box-Cox transformation for experimental replicate 1 (A) and replicate 2 (B). The critical value of enrichment score cutoff at p = 0.05 is indicated by the green horizontal line. The R^2^ linear correlation coefficients are also shown on the plots. The Box-Cox lambda values used for transformation of enrichment scores are 0.1033 and 0.1136 for replicate 1 and 2 respectively.

The Box-Cox transformed scores of Cappable-seq data are normally distributed, as shown by Q-Q plots (Fig 2) with R^2^ valuesof 0.9348 and 0.9279 for replicate 1 and replicate 2, respectively. Based on the fitted normal distribution and the conventional p-value threshold of 0.05, the critical value cutoffs of the transformed enrichment score were determined to be 2.82 and 3.31 for replicate 1 and replicate 2, respectively. Using these cutoffs, recall of known transcript 5′ ends was assessed from the significantly enriched positions. We selected 148 TSS positions manually annotated in the RegulonDB database with strongly supported evidence for recall tests. For some annotated TSS positions, enrichment values could not be determined owing to the absence of mapped reads, leaving 146 positions for assessing recall. The analysis results are shown in Table 1.

**Table 1:** Cappable-seq data analysis results for ToNER and TSSAR.

	ToNER	TSSAR
Number of mapped reads		
- Enriched library (replicate 1)	13,769,054	
- Enriched library (replicate 2)	6,425,560	
- Unenriched library (control)	18,189,338	
Number of genomic positions with enrichment scores		
- Enriched library (replicate 1)	1,280,972	
- Enriched library (replicate 2)	921,474	
Number of detected enriched positions (p = 0.05)		
- Replicate 1	64,993	85,161
- Replicate 2	40,600	62,719
- Combined p-value from 2 replicates	99,789	102,548
Recall rate* (p = 0.05)		
- Replicate 1	86.99% (127)	69.86% (102)
- Replicate 2	74.66% (109)	76.71% (112)
- Combined p-value from 2 replicates	92.47% (135)	70.55% (103)

* The results are shown in the following format: % detection (number of detected TSSs)

### Comparison with available software for transcript 5′ end detection

Cappable-seq is a recently developed transcriptomic method, and no data analysis tool employing a statistical model has been specifically developed for it. In Ettwiller et al. (Cappable-seq original publication) [4], a non-statistical method using fixed thresholds, which is similar in concept to that used in the TSSpredator algorithm [5], was employed. We did not intend to compare the results generated by ToNER with those reported in [4] since it is outside the scope of our study. The main objective of our study is to identify statistically significant enriched positions based on an automatically adjusted enrichment cutoff suitable for each dataset.

We chose the TSSAR software [7] for performance comparison with ToNER due to the fact that this tool employs a frequentist statistical method to identify significantly enriched read start positions. TSSAR was originally developed for analysis of dRNA-seq data, although to our knowledge, it has not been tested on Cappable-seq data. The recall of annotated TSS positions (p-value cutoff of 0.05) detected by ToNER is higher than TSSAR for replicate 1 (127 vs 102) and slightly lower for replicate 2 (109 vs 112). The recall for combined p-value test is greater than individual experiments for ToNER, in which the number of detected TSSs increased to 135 (92.47%). In contrast, combined p-value test recall is no better than individual experiments for TSSAR (Table 1). The combined p-value test provides greater power for ToNER compared with TSSAR, despite the observation that the total number of enriched positions called significant by combined p-value test for ToNER (99,789) is lower than TSSAR (102,548). This suggests that ToNER is also more specific than TSSAR, although unfortunately there are insufficient sites available that can be considered as true negative unenrichable 5′ ends that can be used for assessing precision.

**Fig 3:**
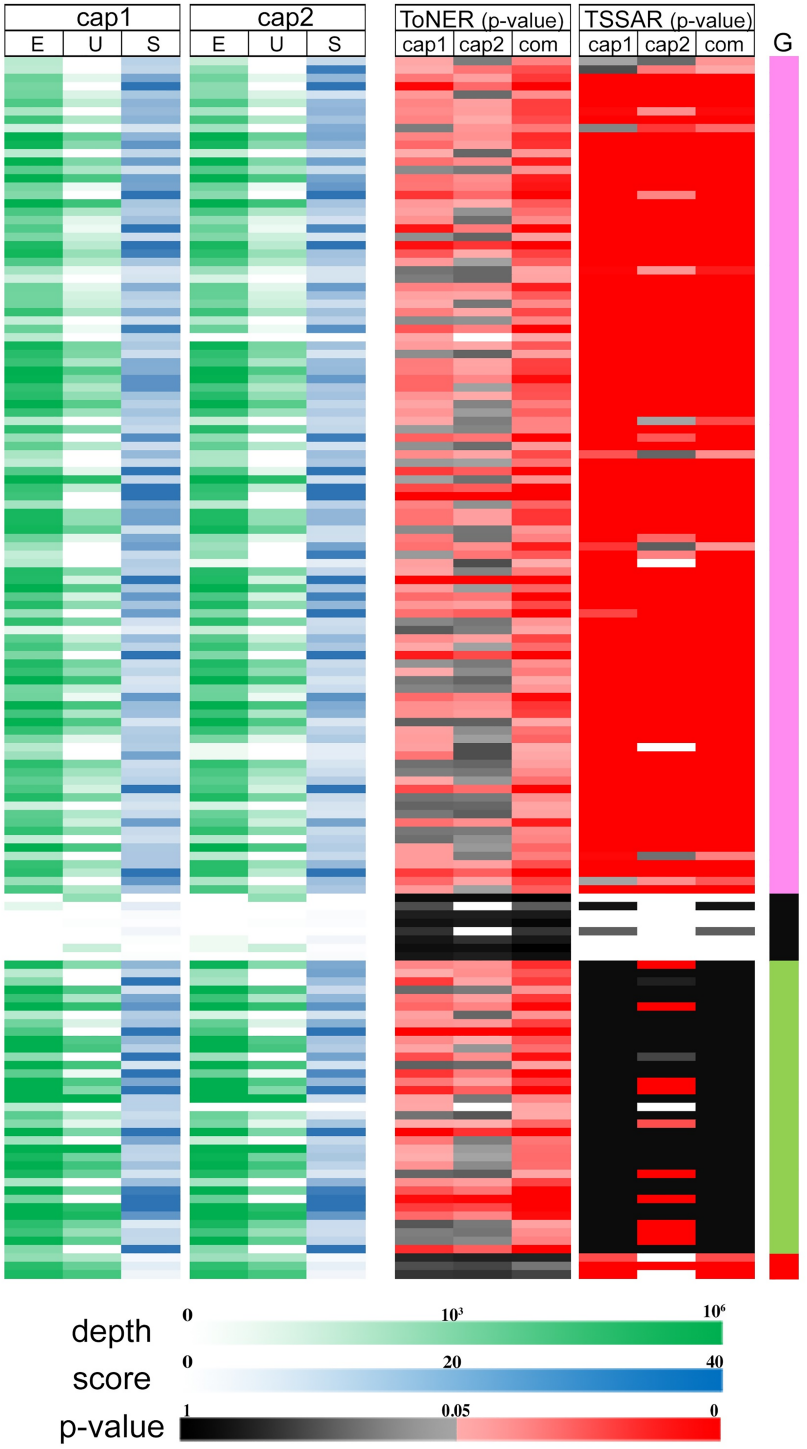
Cappable-seq data and statistics of enrichment reported by ToNER and TSSAR for known transcription start sites (TSS). The Cappable-seq data and statistics of enrichment for 146 known *E*. *coli* TSS annotated in RegulonDB are shown in two parts. The first part on the left displays the normalized read depth as indicated by the green color gradient for the enriched library (E) and unenriched library (U), and the associated ToNER enrichment score (S) as indicated by the blue color gradient for each TSS in the Cappable-seq experimental replicate1 (cap1) and replicate 2 (cap2) datasets. The second part on the right displays nucleotide enrichment p-values obtained from ToNER and TSSAR analyses. For each software, the p-values for replicate 1 (cap1), replicate 2 (cap2), and Fisher’s combined p-values from both replicates (com) are shown. P-values of nucleotide enrichment are indicated in black color gradient for non-significant positions, whereas the significantly enriched positions (p<0.05) are shown in red color gradient. Positions in white have no reported p-value. This can occur because there are no mapped reads, and in case of TSSAR, positions with no statistic can also occur when there are more mapped reads in the unenriched library compared with the enriched library. The annotated TSS positions are grouped based on combined p-values, as indicated by the color bars in the ‘G’ column on the far right: positions detected with significant enrichment (p<0.05) by ToNER and TSSAR (pink); not detected by either software (black); detected by ToNER only (green) and detected by TSSAR only (red).

To explore the possible reasons why ToNER has better recall than TSSAR for the combined p-value test, we examined the analysis results for the known TSS positions in more detail. As shown in Fig 3, p-values of enrichment calculated by ToNER are largely consistent among the two replicates, which explains the increased power to detect when combining p-values across replicates. In contrast, p-values reported by TSSAR are inconsistent between replicates for a number of genomic positions. The disparity of p-values reported by TSSAR is unexpected, since enriched read counts are highly correlated between the Cappable-seq experimental replicates, as shown in the original publication [4].

**Fig 4:**
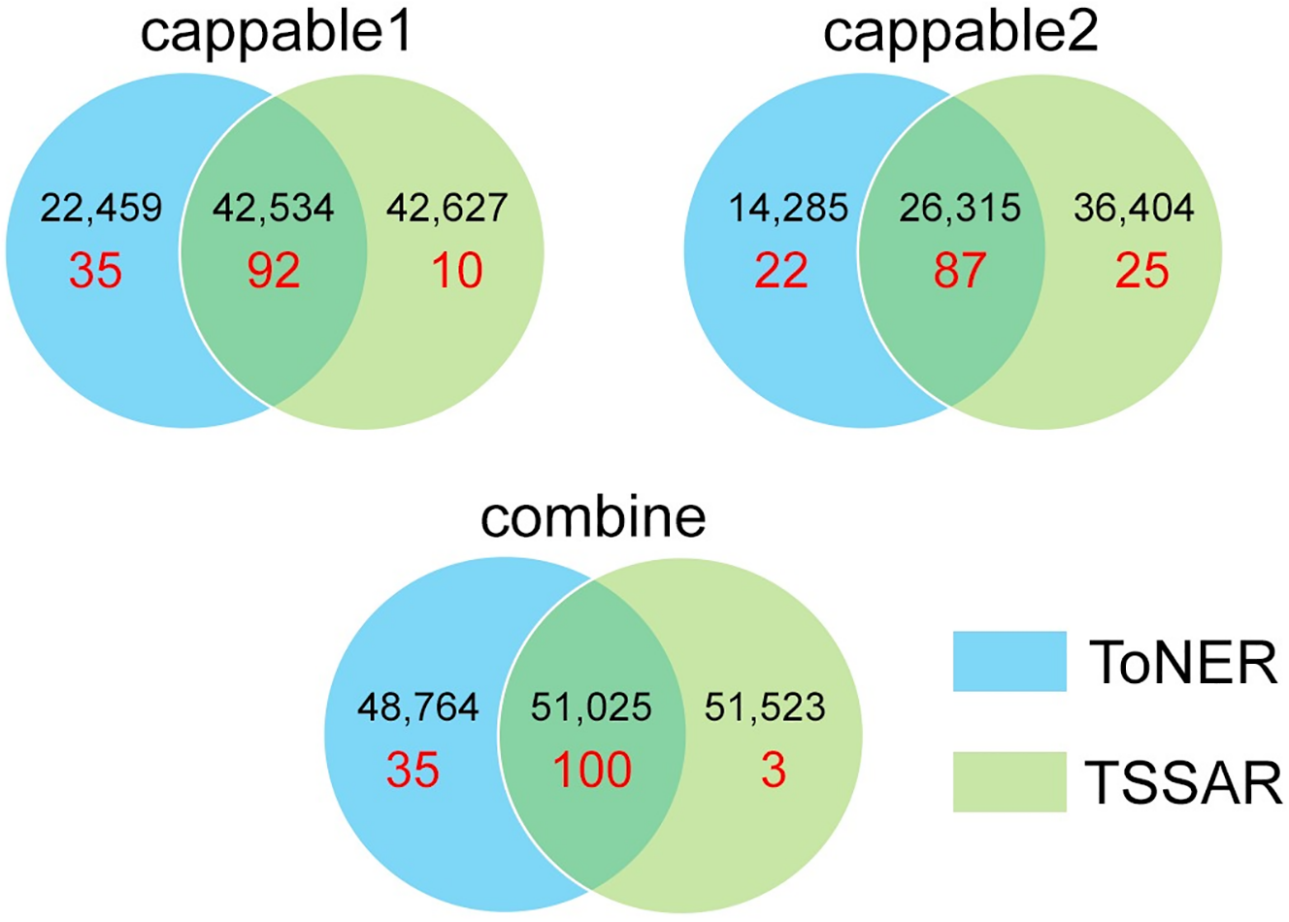
Comparison of significantly enriched nucleotides detected by ToNER and TSSAR. Venn diagrams show overlaps of nucleotides detected as significantly enriched (p<0.05) from ToNER and TSSAR analyses for replicate 1 (cappable1), replicate 2 (cappable2), and the Fisher’s combined results from the two replicates (combine). The number of whole transcriptome significantly enriched positions are shown in black, whereas the number of enriched positions corresponding to known transcription start sites annotated in RegulonDB are shown in red.

The majority of annotated TSS positions were detected as significantly enriched from the Cappable-seq data by both ToNER and TSSAR tools. However, some positions (including annotated TSS) were detected in one tool but not the other (Fig 4). We explored the discrepancy of annotated TSSs detected as significant enriched positions between the two programs. For Cappable-seq replicate 1, all 35 TSSs which can be identified by ToNER but not TSSAR are located in regions that could not be modeled with zero-inflated Poisson regression (total 253 unmodeled regions). For replicate 2, 132 genomic regions could not be modeled by TSSAR, which included 24 annotated TSSs. 21 of these TSSs in unmodeled regions were detected as significantly enriched positions by ToNER. Most of the TSSs detected by ToNER but not by TSSAR have abundant read counts in the enriched library (Fig 3). For the combined p-value tests, the three TSSs identified as significant by TSSAR but not by ToNER have low enrichment scores (<6).

### Cappable-seq data analysis with different software settings

The reporting of transcript 5′ ends with significant enrichment from Cappable-seq data was assessed using different ToNER parameter settings. The tested parameter settings included: analysis method (fitted distribution or percentile rank); minimum read depth of analysed positions (1, 2, and 3); the inclusion of positions with mapped reads from either (pseudo-count default) or both enriched/unenriched libraries (non pseudo-count setting), and different meta-analysis methods between replicates (combined p-value, union, and intersection of results) in comparison with individual replicate. The enrichment score critical value cutoffs calculated from the fitted normal distribution and 95^th^ percentile rank approaches under the default setting (with minimum read depth of one) are quite close and the recall rates of annotated TSS are similar (S1 Table). The effect of filtering read depth was tested, including filtering based on total read depth, and the non pseudo-count option in which positions lacking read depth in either library were also discarded. Recall rates declined overall with these filterings due to higher score cutoffs, and the disparity of recall rates between fitted normal distribution and percentile rank methods increased with the degree of filtering for the default pseudo-count option (S1 Table). It should be noted that recall using combined p-values from replicate experiments was higher under all filtering settings compared with the percentile rank method (S1 Table), highlighting the increased power to detect by statistical modeling.

### Application of ToNER for transcript 5′ end detection from dRNA-seq data

Although we developed the ToNER tool for newer, more efficient RNA-seq enrichment techniques without specific statistical analysis mehods such as Cappable-seq, we tested ToNER for analysis of data from the older and less efficient dRNA-seq method. dRNA-seq datasets from *E*. *coli* grown under different conditions [13] were analysed by ToNER (S2 Table). The dRNA-seq data can be transformed and fitted to the normal distribution, with the exception of the biological replicate no. 2 from cells grown in LB medium and harvested at OD = 0.4, in which the fit to normal distribution was just below the acceptable value of R^2^ cutoff. The lower efficiency of enrichment employed in dRNA-seq is reflected in the enrichment score critical value cutoff, which is lower for dRNA-seq (range 2.9–10.1 before transformation) compared with Cappable-seq (11.9 and 16.6 before transformation for replicate 1 and 2, respectively).

**Fig 5:**
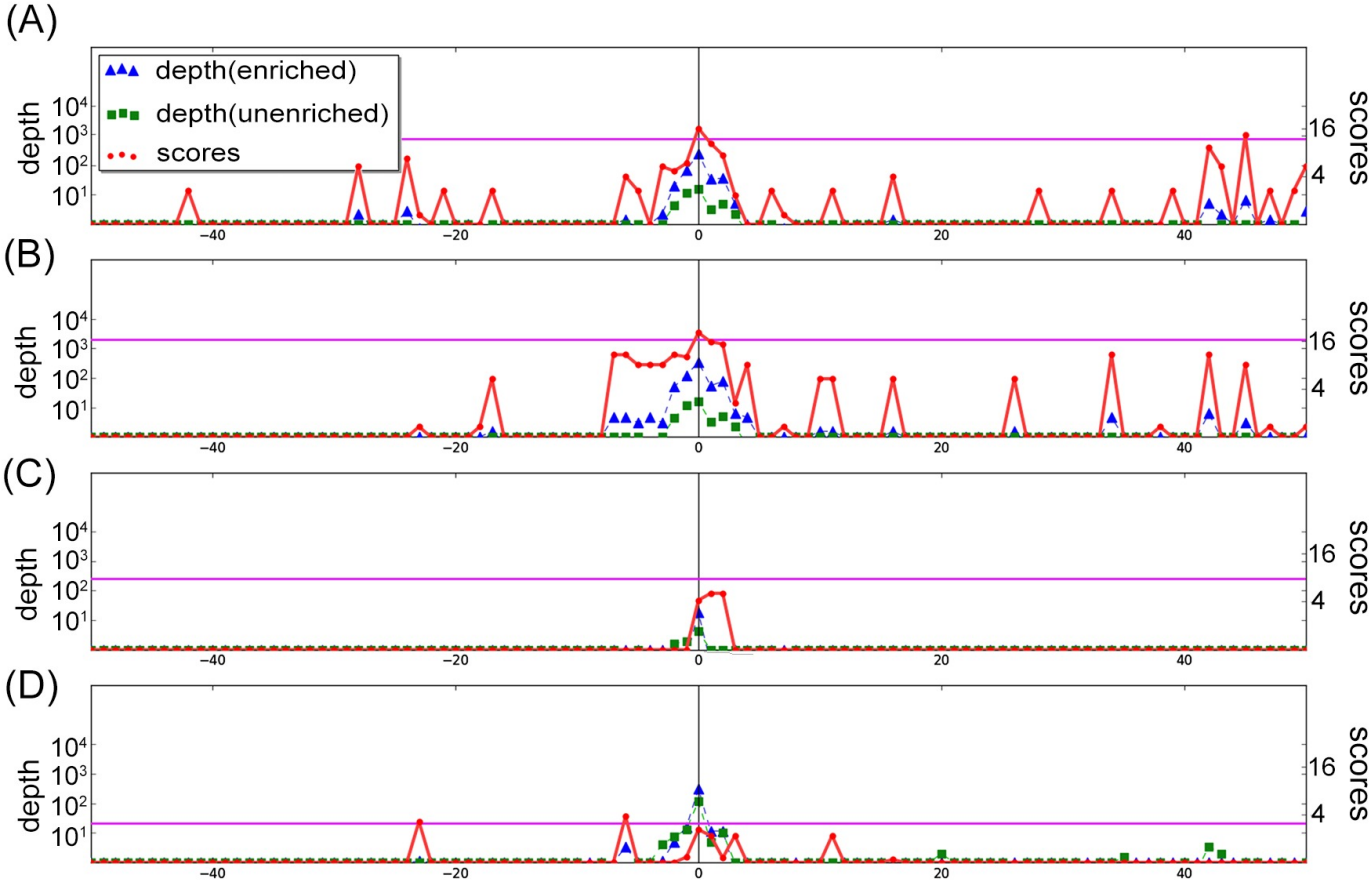
Example of an annotated TSS detected by ToNER from Cappable-seq but not dRNA-seq data. The plots of normalized read depth values of enriched and unenriched libraries including the corresponding enrichment scores reported by ToNER are shown for the 100 bp window (from -50 bp upstream to +50 bp downstream) of an annotated TSS position of *E*. *coli* (NC_000913.2 position 4,271,956; ‘-‘ strand). Data from the Cappable-seq protocol [4] are shown for Cappable-seq replicate 1 (A) and Cappable-seq replicate 2 (B). Data from the dRNA-seq protocol [13] are shown for dataset M63_0.4_B1_L1_GA (C) and dataset M63_0.4_B2_L1_HS2 (D). The ToNER calculated p-values of the annotated TSS position reported in Cappable-seq replicate 1, replicate 2, and combined result are 0.0262, 0.0327, and 0.0017, respectively. For dRNA-seq, the p-values reported in replicate B1, replicate B2, and combined result are 0.1356, 0.0726, and 0.0211, respectively.

Inspection of the transcript 5′ ends identified by ToNER for the dRNA-seq datasets showed that the recall of annotated TSS was lower compared with Cappable-seq datasets. Recall of annotated TSS among the dRNA-seq datasets was higher using the TSSAR program (which was specifically designed for data of this type) compared with ToNER (S2 Table). The lower recall of TSS by ToNER for dRNA-seq data is due to the low efficiency of enrichment used in the dRNA-seq method, which makes it more difficult to distinguish TSS from non-enriched positions using a global model of enrichment distribution. To illustrate this, experimental data are shown for an annotated TSS in Fig 5. Two datasets of *E*.*coli* dRNA-seq reported in Thomason et al. [13] (M63 0.4 B1 and B2 datasets), were selected for demonstration since the growth conditions used in these experiments are most similar to Ettwiller et al. [4], i.e. minimal growth medium and the same carbon source of 0.2% glucose. The ToNER tool could detect this TSS as signifcantly enriched in both Cappable-seq experiments and after combining p-values. In contrast, ToNER could not detect this TSS individually from either of the two dRNA-seq experiments. The weak, but consistent enrichment of this TSS in the dRNA-seq experiments could, however, be detected as significant by ToNER after combining p-values. The TSSAR tool could detect this TSS as significantly enriched among individual dRNA-seq and Cappable-seq datasets, suggesting that the local statistical modeling used by TSSAR can be more sensitive than ToNER for detecting weak enrichment signals in individual experiments.

**Fig 6:**
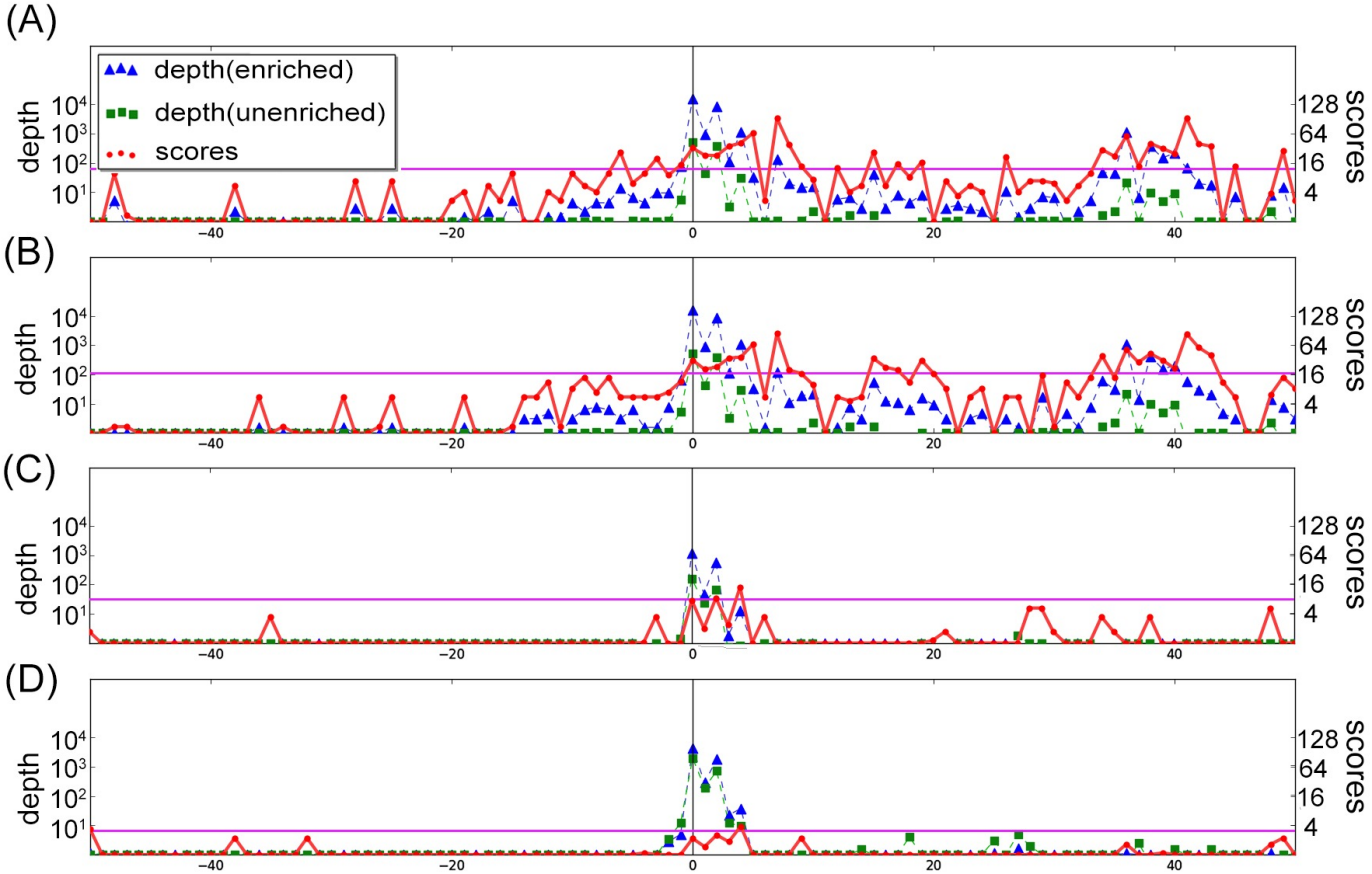
Example of an annotated TSS which is located in an unmodeled region by TSSAR in both Cappable-seq and dRNA-seq datasets. The plots of normalized read depth values of enriched and unenriched libraries including the corresponding enrichment scores reported by ToNER are shown for the 100 bp window (from -50 bp upstream to +50 bp downstream) of an annotated TSS position of *E*. *coli* (NC_000913.2 position 3,309,808; ‘-‘ strand). Data from the Cappable-seq protocol [4] are shown for Cappable-seq replicate 1 (A) and Cappable-seq replicate 2 (B). Data from the dRNA-seq protocol [13] are shown for dataset M63_0.4_B1_L1_GA (C) and dataset M63_0.4_B2_L1_HS2 (D). The ToNER calculated p-values of the annotated TSS position reported in Cappable-seq replicate 1, replicate 2, and combined result are 0.0043, 0.0126, and 0.0017, respectively. For dRNA-seq, the p-values reported in replicate B1, replicate B2, and combined result are 0.0549, 0.1046, and 0.0125, respectively.

Although TSSAR in general has greater power to detect TSS positions from dRNA-seq data than ToNER, TSSAR still fails to detect some TSS positions which are located in unmodeled regions. An example of unmodeled region is illustrated in Fig 6. This annotated TSS region was unmodeled by TSSAR from both Cappable-seq and dRNA-seq data. ToNER can detect this TSS as a signifcantly enriched position individually in both replicates of Cappable-seq data. Although not quite reaching significance in individual dRNA-seq datasets, ToNER can detect this TSS as significantly enriched after combining p-values from experimental replicates.

### Other potential applications of ToNER

ToNER can be used to analyse other types of RNA-seq data comprising enriched/control pairs for the purpose of annotating nucleotides significantly enriched for a biological feature of interest. A wide variety of RNA modifications are believed to regulate structural and catalytic functions of RNAs, of which *N*^6^-methyladenosine (m^6^A) is the most common internal RNA modification found in eukaryotes [1].

We analysed human m^6^A-enriched and unenriched control RNA-seq data generated using the m^6^A-seq protocol [15]. Since m^6^A sites can be present at any position in a transcript, the ToNER tool was implemented using the option of analysing all read positions and the default pseudo-count setting. The transformed enrichment scores fit well to the normal distribution, as shown by R^2^values of 0.9245 and 0.9296 (S2 Fig). We found that 10,116,065 and 6,977,037 sites passed the enrichment score cutoff at p = 0.05 for replicates 1 and 2, respectively. Interestingly, more than half of the union enriched positions of two replicates (54%) were located outside of annotated genes. Of the enriched positions outside of annotated genes, 57,383 positions showed strongly enriched signals with high read depth (total reads > 100) in both replicates. These positions were not considered in the original paper, which limited the search to annotated genes [15]. Since m^6^A sites can be present at any position on the enriched RNA fragments, peak finding is needed to identify the precise location of m^6^A nucleotides.

The analysis of m^6^A-seq data is provided as an example for application of the ToNER software in eukaryotes. In this application, ToNER provides an initial list of nucleotides with significantly enriched signals which users can use as an input for a peak finding algorithm to locate m^6^A sites more precisely. Since ToNER explores the whole genome, it can be used to complement existing tools [17, 18] for identifying m^6^A sites, which limit the search to annotated genes.

The m^6^A-seq data are more complex than Cappable-seq data, considering the number of genomic positions included in statistical modeling of score distribution of m^6^A-seq data is about 100 times greater than that of Cappable-seq data. From the results, we conclude that ToNER could be applied to the more complex datasets of eukaryotic species. It should be noted that users must ensure that sufficient random access memory (RAM) is available. RAM can be limiting for analysis of complex transcriptomes, e.g. mammalian and when all read positions are included in the analysis.

## Discussion

The ToNER software tool presented here offers a conceptually simple approach for identifying genomic positions of biological interest from RNA-seq data in which an enrichment strategy is employed. The software was designed to analyse data from experimental RNA enrichment protocols that generate enriched and unenriched (control) library datasets, especially recently developed techniques without a customized analysis method.

We have demonstrated the applicability of ToNER software to the TSS identification problem, in which prokaryotic TSS can be reliably detected from Cappable-seq data. All available nucleotide positions with enrichment scores are used by ToNER to determine the global distribution and the significance cutoff. Our approach contrasts with the local model fitting approach used by other tools for statistical analysis of enriched RNA-seq data such as TSSAR. The separation of the genome into regions for modeling by TSSAR is necessary since transcripts vary in abundance, and thus read count distributions. However, the appropriateness of a modeled read count distribution is strongly dependent on the chosen window size of analysed genomic region [7]. If the region is too large, it may encompass more than one transcript and 5′ ends of lower abundance transcripts may not be detected owing to the greater variance. On the other hand, if the region is too small, the data will be insufficient for modeling and no enrichment statistic will be reported in that region. Therefore, the enrichment statistics reported by the TSSAR program may be less reliable for some regions than others.

Experimentally verified TSS positions were identified by the ToNER tool from Cappable-seq data with a high recall rate. We found known TSS residing in regions that cannot be modeled by TSSAR, despite an abundance of mapped reads in the Cappable-seq enriched library dataset. Therefore, the Poisson modeling approach used in TSSAR may not be well suited for analysing data from efficient enrichment methods such as Cappable-seq owing to the problem of missed identifications in unmodeled regions. Furthermore, although the local modelling approach used by TSSAR has a greater power to detect TSS for dRNA-seq data than ToNER, some TSS are still missed owing to their locations in unmodeled regions. ToNER can thus be used to complement TSSAR for analysis of dRNA-seq data for providing the most comprehensive annotation of TSS.

The statistical modeling of enrichment scores used by ToNER is rather conservative in the sense that some TSS positions with low enrichment scores were not detected from the Cappable-seq data. However, the markedly increased recall rate of combined results from two replicates using Fisher’s combined probability test available in ToNER highlights the consistency of results among replicates obtained from the software and effective use of the available data, allowing us to detect weakly enriched sites. As sequencing costs fall, future studies of RNA-seq with enrichment will likely include more replicates, which provide increased power to detect biologically relevant features. With sufficient replicates and sequencing depth, it may be possible to detect enriched features even from low abundance transcripts.

The list of significantly enriched positions provided by ToNER requires careful interpretation for biological relevance. First, it should be realized that annotation of functionally relevant nucleotides is limited by the sampled material, such that the study must be designed to include sufficient biological replicates. Furthermore, replicate samples must be enriched independently to satisfy the assumption used for statistical meta-analysis for maximizing power to detect enriched nucleotides. Although statistical meta-analysis may help in eliminating potential false positive nucleotides that are not consistently enriched across experimental replicates, the study could be designed with internal controls for assessing precision. If no true negative RNA species are known to be present in the sample, then artificial RNAs synthesized in vitro lacking the feature of interest, known as “spike-ins” [19, 20] could be added to the sample before enrichment. The enrichment scores of the spike-ins could then be used to determine the false positive rate at different p-value cutoffs, and thus control false discoveries.

Filtering is typically employed in RNA-seq experiments to exclude transcriptional noise and improve accuracy when characterising low abundance transcripts. We found that filtering did not markedly improve the goodness of fit of enrichment scores, and also reduced sensitivity to detect known 5′ ends. Therefore, we do not think that initial filtering of read depth for model fitting is beneficial for our approach. However, transcriptional noise could be reduced by applying a filter of minimum read depth to positions called significant by ToNER using the information provided in the reported output files. If data from spike-ins are available (see above), the read depth filter could be tuned according to the false-positive rate determined from the spike-ins. Moreover, in addition to read depth filtering, it may be useful to cluster significantly enriched positions located in close proximity to one another to account for variation caused by biological (e.g. alternative TSS) and technical (e.g. sample degradation during library preparation) factors. We cannot suggest a general scheme for clustering, since the appropriate clustering method depends on the data analysed and the biological research question. Various clustering tools are available for different purposes, for example, a script for TSS clustering was provided in [4].

We have also applied ToNER for identifying significantly read-enriched positions from human m^6^A-seq data and showed that the data can be modeled by our approach. Users can use ToNER to identify significantly enriched positions, which could be processed further by available peak finding tools to obtain m^6^A sites. The ExomePeak [18] and MeTPeak [17] tools were developed to improve the detection of enriched sites from m^6^A-enriched/control paired data. These tools apply local Poisson modeling to genomic regionsin a similar way to TSSAR. Instead of dividing the genome into arbitrary blocks of equal length as in TSSAR, ExomePeak and METPeak use available exome annotations to define boundaries of regions for Poisson modeling. These algorithms thus can only be used for well-annotated genes, and cannot be used to identify features in novel genes, or in organisms with poorly annotated genomes. ToNER allows analysis of all genome positions; therefore, potential m^6^A sites can be identified outside of annotated gene regions.

We envision that ToNER could potentially be used to analyse a variety of enrichment datasets. ToNER can be parameterized with various options to match with the users’ needs. For example, the enrichment analysis could be selected to focus on read start, read end, or all positions of reads, according to the enrichment method used. The variation in the efficiency of enrichment among experimental replicates is accounted for in our approach, in which the critical value of significant enrichment is determined separately for each experimental dataset. Moreover, since statistics are reported for all nucleotides, combined statistics across replicates can increase power to detect enriched features.

## Conclusions

We introduce a new software called ToNER, that can identify enriched sites from differential RNA-seq experiments comprising enriched and unenriched libraries. We have demonstrated that ToNER can be used to identify TSS from Cappable-seq data in prokaryotes. It could also be applied to more complex data of eukaryotes such as m^6^A-seq to locate enriched positions for further analysis. ToNER has many flexible options, such as adjustable enrichment score cut-off and meta-analysis which can be used to improve detection power when experimental replicates are available.

## Supporting information

S1 Fig Graphical analysis outputs of ToNER software for Cappable-seq datasets. (A) Density plot of number of read starts per position for the enriched library (Library 1) and unenriched library (Library 2). (B) Pie chart showing the fraction of genomic positions with the presence/absence of mapped reads in paired libraries. The pencentages of genomic positions with mapped reads found in both paired libraries (both), only in enriched library (only_1), and only in unenriched library (only_2) are shown with the number of corresponding positions shown in parentheses.
(TIF)

S2 Fig The normal Q-Q plots of the calculated enrichment scores from m^6^A-seq data. Q-Q plots of enrichment score quantiles calculated from m^6^A-seq data (vertical axes) versus normally distributed theoretical quantiles (horizontal axes) are shown for scores before and after Box-Cox transformation for experimental replicate 1 (A) and replicate 2 (B). The critical value of enrichment score at p = 0.05 is indicated by the green horizontal line. The R^2^ linear correlation coefficients are also shown on the plots. The Box-Cox lambda values used for transformation of enrichment scores are -0.2397 and -0.1226 for replicate 1 and 2 respectively.
(TIF)

S1 Table ToNER analysis results of Cappable-seq using different software settings. This file includes the analysis results of *E*.*coli* Cappable-seq data using the default (pseudo) and non-pseudo option. The result statistics, model parameters, and recall rates for different settings of read filtering were displayed.
(XLSX)

S2 Table ToNER analysis parameters of all datasets. This file includes the result statistics, model parameters, and recall rates of all datasets (Cappable-seq, m^6^A-seq, dRNA-seq) analyzed using the default settings.
(XLSX)
