# Supplementary figures and images for: ToNER: A tool for identifying nucleotide enrichment signals in feature-enriched RNA-seq data
Source: PLoS One. 2017 May 25;12(5):e0178483. doi: 10.1371/journal.pone.0178483 (PMC5444824; doi:10.1371/journal.pone.0178483)

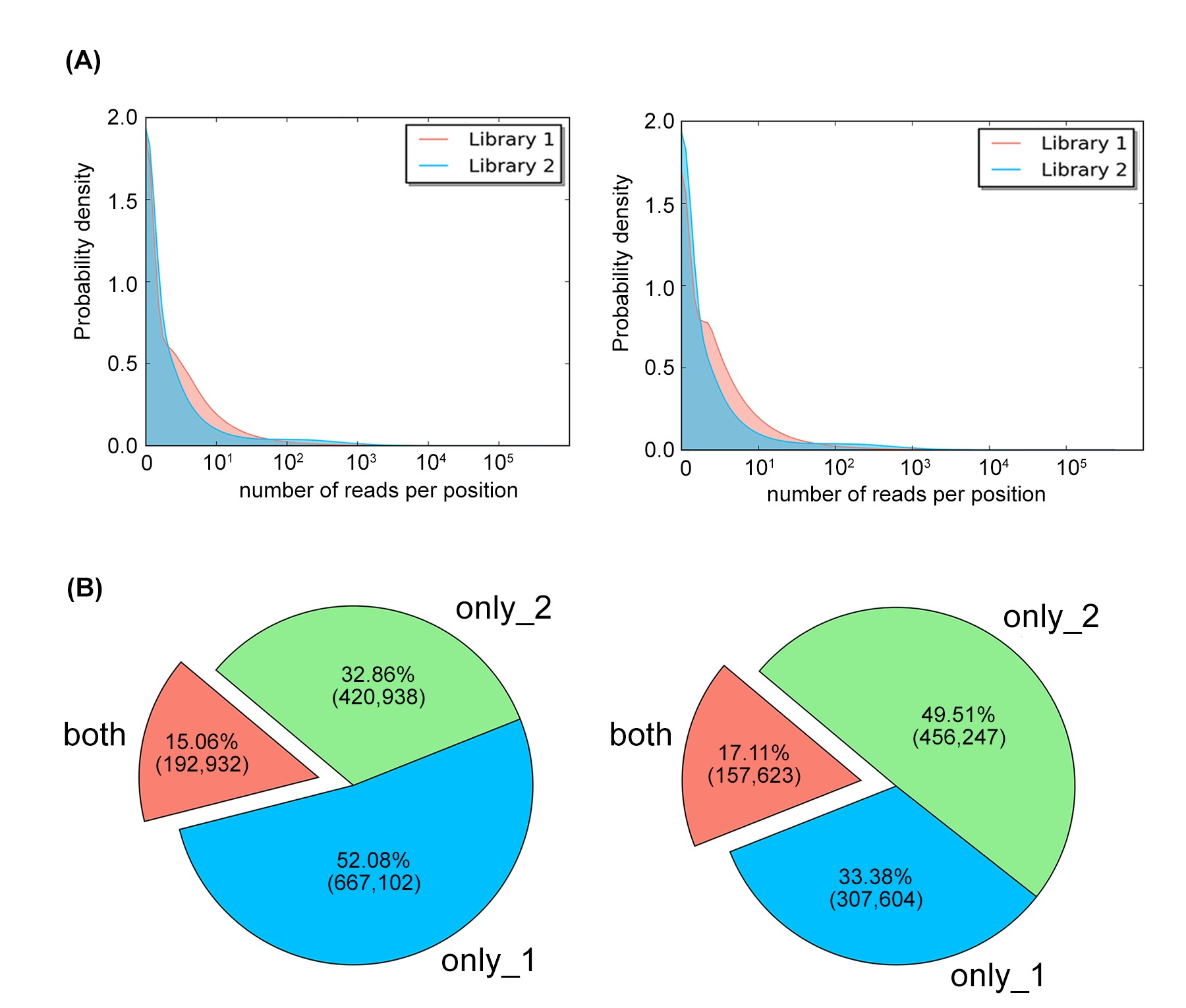

Supplement: S1 Fig — (A) Density plot of number of read starts per position for the enriched library (Library 1) and unenriched library (Library 2). (B) Pie chart showing the fraction of genomic positions with the presence/absence of mapped reads in paired libraries. The pencentages of genomic positions with mapped reads found in both paired libraries (both), only in enriched library (only_1), and only in unenriched library (only_2) are shown with the number of corresponding positions shown in parentheses. (TIF) [file pone.0178483.s001.tif]

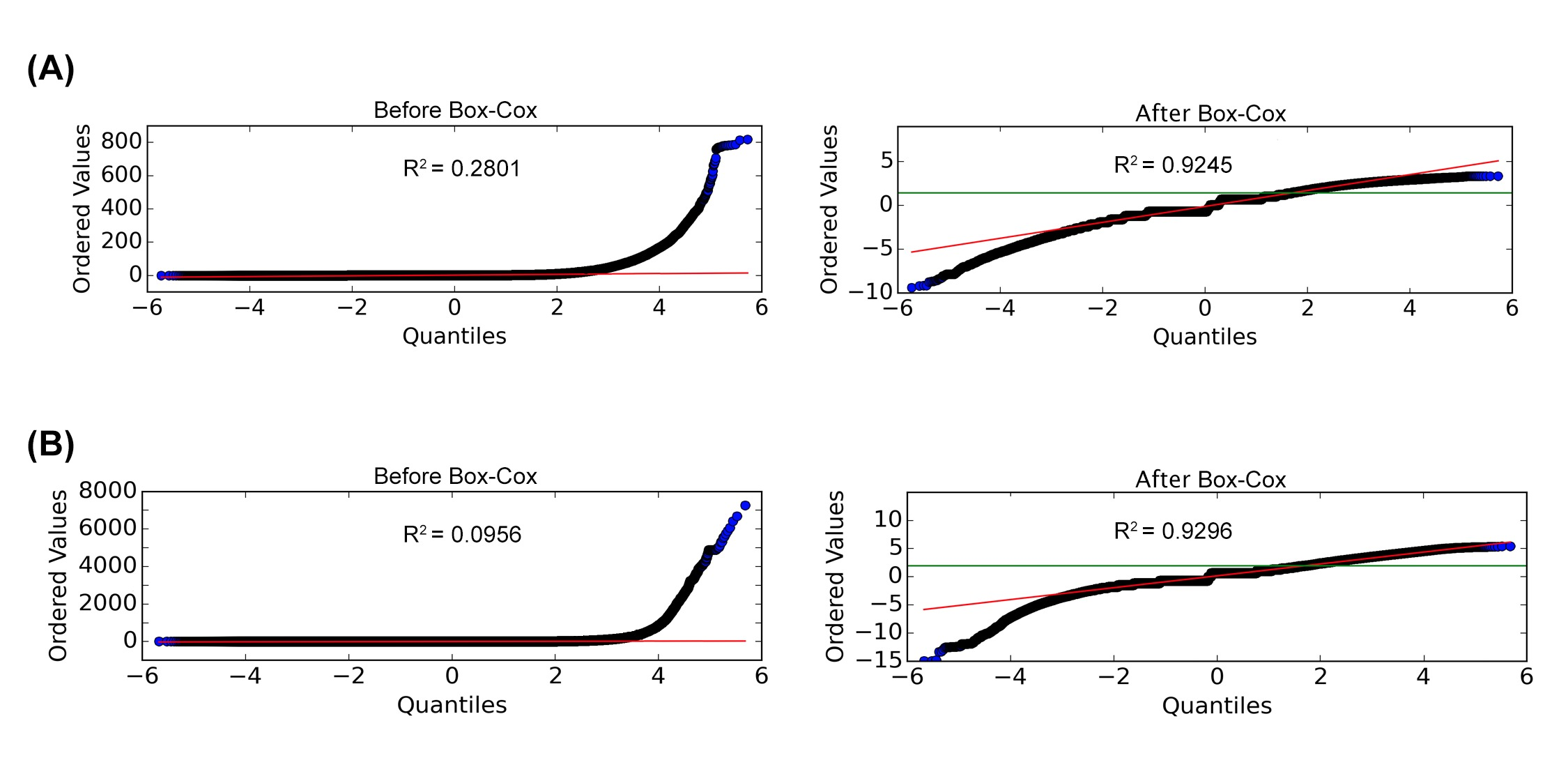

Supplement: S2 Fig — Q-Q plots of enrichment score quantiles calculated from m6A-seq data (vertical axes) versus normally distributed theoretical quantiles (horizontal axes) are shown for scores before and after Box-Cox transformation for experimental replicate 1 (A) and replicate 2 (B). The critical value of enrichment score at p = 0.05 is indicated by the green horizontal line. The R2 linear correlation coefficients are also shown on the plots. The Box-Cox lambda values used for transformation of enrichment scores are -0.2397 and -0.1226 for replicate 1 and 2 respectively. (TIF) [file pone.0178483.s002.tif]
